# The Atomic Observation of the Structural Change Process in Pt Networks in Air Using Environmental Cell Scanning Transmission Electron Microscopy

**DOI:** 10.3390/nano13152170

**Published:** 2023-07-26

**Authors:** Masaki Takeguchi, Toshiaki Takei, Kazutaka Mitsuishi

**Affiliations:** National Institute for Materials Science, 1-2-1 Sengen, Tsukuba 305-0047, Japan; takei.toshiaki@nims.go.jp (T.T.); mitsuishi.kazutaka@nims.go.jp (K.M.)

**Keywords:** scanning transmission electron microscopy, environmental cell, Pt network, atomic resolution, catalyst, grain, grain boundary, atomic diffusion

## Abstract

The structural change in Pt networks composed of multiple chain connections among grains was observed in air at 1 atm using atomic-resolution environmental cell scanning transmission electron microscopy. An aberration-corrected incident electron probe with a wide convergence angle made it possible to increase the depth resolution that contributes to enhancing the signal-to-noise ratio of Pt network samples in air in an environmental cell, resulting in the achievement of atomic-resolution imaging. The exposure of the Pt networks to gas molecules under Brownian motion, stimulated by electron beams in the air, increases the collision probability between gas molecules and Pt networks, and the Pt networks are more intensely stressed from all directions than in a situation without electron irradiation. By increasing the electron beam dose rate, the structural change of the Pt networks became significant. Dynamic observation on an atomic scale suggested that the structural change of the networks was not attributed to the surface atomic-diffusion-induced step motion but mainly caused by the movement and deformation of unstable grains and grain boundaries. The oxidized surface layers may be one of the factors hindering the surface atomic step motion, mitigating the change in the size of the grains and grain boundaries.

## 1. Introduction

The search for high-performance and long-lasting catalysts remains a critical issue for developing electrochemical-based devices such as water electrolysis cells, fuel cells, and rechargeable batteries [1,2,3], which require improvements in power density and stability. The mass activity of catalysts can be enhanced by increasing the specific activity and active surface area. Using precious metal nanoparticles supported by carbon-based materials is the most common approach in developing the above catalysts. However, under reactive environments, carbon supports are likely to be damaged, and nanoparticles often cannot maintain their shape, size, and positions, resulting in the active surface area decreasing in the short term [4,5]. Furthermore, high costs and supply limitations are associated with precious metals, especially Pt-group metals. Therefore, precious metal alloys with more earth-abundant materials, including metal-organic frameworks, core–shell structures, and high-entropy alloys, have been investigated to not only reduce the amount of precious metal but also to make them resistive to the reactive environment [6,7,8,9]. The improvement in stability has also become more crucial, and various strategies have been proposed. Bulk-like nanoarchitectures with high surface area, such as nanopores, nanosheets, and self-supporting networks, are promising in terms of both stability and activity, leading to long-lasting high-performance catalysts [10,11,12,13,14,15,16,17]. However, catalytic reactions still cause structural changes in their nanostructures, followed by catalytic deactivation, limiting the catalysis lifetime. For instance, in the case of nanoporous catalysts, coarsening of the nanoporous structure occurs during the catalytic reaction, and its performance gradually worsens during the process [17]. The degradation mechanism of network-structure catalysts is considered to be similar.

The coarsening process of nanopores in nanoporous gold (NPG) in an environment of 1 vol% CO/air with a pressure range of 1–30 Pa at room temperature was previously investigated on an atomic scale using high-resolution environmental transmission electron microscopy (TEM) [17]. The NPG with complex bicontinuous porous structures was fabricated by dealloying an Ag_65_Au_35_ alloy leaf. The average diameter of both the gold ligaments and nanopores was approximately 30 nm. It was revealed that the coarsening of the nanopores and the thinning of the ligaments were attributed to the rapid diffusion of gold atoms at surface steps and the surface segregation of residual silver atoms. Because no significant structural change of the NPG sample was observed in heating experiments up to 300 °C under a vacuum in TEM, it was concluded that the CO/air catalytic reaction may induce gold atom diffusion and silver atom segregation. Moreover, high-resolution in situ tensile TEM observations of the deformation and fracture processes of NPG ligaments showed that the combination of dislocation plasticity and stress-driven surface atom diffusion can promote the plastic instability of ligaments, finally resulting in the brittle failure of the nanoporous structures [18]. Although the results of gas-environmental and tensile in situ TEMs might be locally limited phenomena, they help clarify what might happen in nanoporous and network-structure catalyst architectures under practical conditions. 

In the catalytic nano-architectures, thermal stress causes local mechanical stress on all parts from all directions. NPG has intrinsically robust ligaments because of their relatively large diameter, and therefore the degradation mechanism is mainly attributed to nanopore coarsening and ligament thinning, promoted by atomic diffusions, as described above. In contrast, network-structure catalysts are predicted to be more fragile because they are composed of multiple chain-like connections of grains and grain boundaries (GB) containing many defects, with sizes smaller than those of NPG ligaments. Nevertheless, it has been reported that self-supporting network-structure catalysts can maintain the initial activity longer than the nanoparticles on supports [9,13]. However, the atomic structural change mechanism of the network catalysts under a reactive environment has rarely been investigated. 

In the present work, the structural change dynamics of Pt networks in an oxidation environment of air at 1 atm and room temperature were observed using environmental cell (EC) scanning TEM (STEM) with an atomic resolution. An aberration-corrected STEM probe with a convergence semi-angle of 28 mrad provided a small depth of the field, thereby dramatically improving the signal-to-noise ratio (SNR) of the Pt networks in an EC, resulting in the amelioration of the STEM resolution. The Pt networks were composed of chain-connected grains and GBs and were deformed during STEM observation. Under electron beam irradiation, primary electrons collide with gas molecules in air and transfer momentum to them, stimulating their Brownian motion. Thus, the collision probability between gas molecules and Pt networks increases, and the Pt networks are more intensively stressed from all directions than the situation without electron irradiation. The present STEM observations showed no remarkable change in size in the grains and GBs, while unstable grains moved and atoms fluctuated at the GBs and surfaces. Moreover, the surfaces were covered with oxide layers. Based on these results, the factors that influence the structural stability of the Pt network in the oxidation environment were considered.

## 2. Materials and Methods

### 2.1. Sample Preparations in Environmental Cells

**Figure 1:**
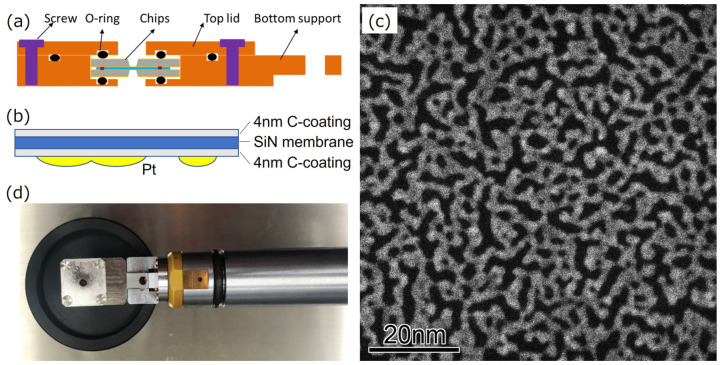
(**a**) Schematic illustrations showing the cross-section of the EC. (**b**) The geometry of Pt network samples, a silicon nitride membrane, and carbon-coated layers. (**c**) HAADF-STEM image of Pt networks in air. (**d**) Photo of the EC attached to a TEM holder.

Figure 1a shows a cross-section of our custom EC, which was assembled by sandwiching a pair of 6 mm × 6 mm Si chips with an electron-transparent silicon nitride membrane window of 30 μm × 300 μm in size [19,20,21]. A spacer of SiO_2_ with a height of 200 nm was deposited on the bottom chip. The thickness of the silicon nitride windows was measured to be approximately 30 nm with a cross-sectional TEM of a specimen that was produced from another Si chip using the focused ion beam lift-out process. Amorphous carbon with 4 nm thickness was sputter-coated on both sides of the Si chips using a precision etching coating system (Model 682, Gatan, Pleasanton, CA, USA). Next, Pt was deposited on the inner side of the top chip using an auto fine coater (JFC-1600, JEOL, Tokyo, Japan), as shown in Figure 1b. Through deposition, Pt nanoparticles formed and then coalesced with each other, finally resulting in the formation of network structures composed of chain-connected grains, as shown in Figure 1d. The image is a high-angle annular dark-field (HAADF) STEM image, and the image intensity of the Pt networks is almost the same throughout, except for in some regions. This is because the network structure was grown two-dimensionally on the silicon nitride membrane surface. The brighter areas correspond to three-dimensional shaped structures, but their numbers are small. After the Pt deposition, the top chip was placed on the bottom chip in air so that the rectangular silicon nitride windows of the top and bottom chips could be orthogonally arranged to obtain a 30 μm × 30 μm square viewing field. Finally, the EC was attached to a single-tilt TEM holder (EM-21010 (SCSH), JEOL, Japan) to complete an EC-TEM holder, as shown in Figure 1c.

### 2.2. Environmental Cell Scanning Transmission Electron Microscopy

The EC-TEM holder was introduced into an aberration-corrected transmission electron microscope (JEM-ARM200F, JEOL, Japan) equipped with a Schottky field emission gun. First, the XYZ position of the viewing field of a 30 μm × 30 μm window was memorized, and then the EC-TEM holder was removed. Next, another TEM holder in which a conventional carbon film-supported Cu microgrid was mounted was introduced into the microscope, and the electron-optics system and aberration correctors were finely tuned. Then, the EC-TEM holder was again introduced into the microscope, the viewing field position was recalled, and the STEM imaging was performed. An aberration-corrected beam probe with an energy of 200 keV and a current of 52.5 pA passing through a condenser aperture (28 mrad convergence semi-angle) was used. The STEM resolution and dwell time were 512 × 512 pixels and 5.0 µs/pixel, respectively. STEM movies were recorded using a high-definition video function in the Gatan Microscopy Suite^®^ (Gatan, USA) with a rate of 10 frames/s.

### 2.3. Achievement of Atomic Resolution by Improving the Depth Resolution of a STEM Probe 

During the STEM observation of samples in an EC, the materials of the two membrane windows and the media packed in the cell deteriorate the incident electron beam probe shape. The lateral spatial resolution of EC-STEM is given as follows [22,23,24]:

(1) $$∆d=\sqrt{{d_{o}}^{2}+{d_{SNR}}^{2}+{d_{blur}}^{2}+{d_{cc}}^{2}}$$

where *d_o_* is the resolution determined by the geometrical optics of an instrument (i.e., it is defined by spherical aberration and a condenser aperture function), *d_SNR_* is the resolution limited by the SNR of an object image, *d_blur_* is the resolution originating from the beam blurring caused by elastic scattering, and *d_cc_* is the resolution limited by chromatic aberration [22,23,24]. Although the geometrical optics and chromatic aberration are fixed by a microscope imaging condition, the influence of the beam blurring of the STEM probe can be minimized when the sample is placed on the inner side of the top membrane. However, the SNR depends on the thicknesses of the membranes and media, which predominantly limit the resolution of EC-STEM [22]. Reducing the membrane thickness to increase SNR is rarely recommended because the robustness of the membrane also decreases. 

The improvement of the depth resolution in STEM (i.e., shortening the elongation of the STEM probe shape along the vertical direction), is the most effective way to enhance the contrast of the sample against the background intensity, i.e., directly relating to the SNR, effectively increasing the resolution in EC-STEM. Since the depth resolution is inversely proportional to a square of the convergence semi-angle in an aberration-corrected probe-forming lens, the wider the convergence semi-angle, the smaller the depth resolution [25,26,27,28,29,30,31]. This technique is especially practical for atomic-sized objects and has been exploited to three-dimensionally visualize atoms on surfaces and inside samples [30,31]. In the present work, an aberration-corrected STEM probe formed with a condenser aperture with a 28 mrad convergence semi-angle was used, for which the depth resolution was theoretically determined to be approximately 3 nm, assuming that the chromatic aberration was negligible. Using this probe, atomic-resolution STEM observations can be performed in air for samples sandwiched between the silicon nitride membranes of an EC. 

### 2.4. The Dose Rate of STEM Observation

The dose rate is a significant factor in environmental electron microscopy because the interaction between incident electrons and materials in the cell (i.e., the sample and its surrounding media) causes damage to the sample and radiolysis of media molecules. The latter generates chemically reactive species such as radicals. This issue requires consideration, in particular, concerning how much of the natural phenomena or structures can be understood from the observed results and how the results should be compensated to clarify the real underlying mechanism or systems. The direct interaction (elastic scattering) between primary electrons and the sample causes knock-on damage when the electron energy is higher than the threshold value of the displacement energy of atoms in the sample. The elastic scattering of electrons by molecules in media under Brownian motion also occurs, and momentum is transferred from electrons to the molecules, accelerating the Brownian motion speed, as previously mentioned. The inelastic scattering of primary electrons results in the emission of secondary electrons from the sample, media molecules, and cell window membranes. Secondary electrons are the main factor in radiolysis, especially in the case of the liquid cell [32]. The radiolysis products are chemically reactive with the sample and often cause unexpected results. Hence, minimizing the dose rate is the general way to mitigate unwanted physical and chemical damage to the samples.

Whereas electrons continuously illuminate a whole viewing area in TEM, in STEM, a finely focused probe is raster-scanned, and each point in the observation area is only exposed for the length of the dwell time during every pass. The dose rate of STEM is described as:

(2) $$Dose\;rate=6.24\times 10^{6}\frac{J}{D^{2}}\;\left[e/{nm}^{2}s\right]=\frac{J}{D^{2}}[pA/{nm}^{2}]$$

where *J* is the beam current, and *D* is the length of one side of the viewing area, assuming that the viewing area is a rectangle. In this study, the STEM beam of 52.5 pA gives a dose rate of the order of 10^−1^ pA/nm^2^, even when magnified 10 million times (enough to achieve atomic-resolution imaging), which is almost the same order as that in conventional high-resolution liquid TEM [33,34]. 

## 3. Results and Discussion

**Figure 2:**
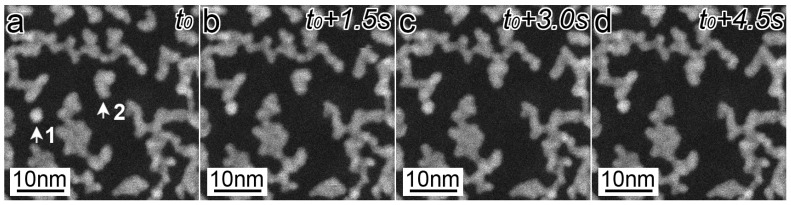
HAADF-STEM images showing the morphology change of Pt networks in air under the dose rate of 3.3 × 10^−2^ pA/nm^2^. (**a**–**d**) were taken every 1.5 s. The islands indicated by white arrows 1 and 2 moved up and finally attached to the neighboring parts.

The morphology change of the Pt networks was small during HAADF-STEM imaging when searching the field of view and focusing at a magnification of less than 1 million times. However, when the magnification was increased to 5 million times, the network structures started transforming, as shown in Figure 2, in which 2a to 2d were taken every 1.5 s under the dose rate of 3.3 × 10^−2^ pA/nm^2^. This gradual transformation was potentially caused by the random attacks of gas molecules and atoms accelerated by electron collisions, rather than the direct electron sputtering, because the transformation speed of Pt networks was dramatically increased when the Pt networks were covered with water in the EC and negligible when they were in a vacuum. It should be noted that the connected parts were more stable than the isolated islands. The islands indicated by white arrows 1 and 2 moved and finally attached to the neighboring parts. The Video S1 shows the gradual change of the whole morphology of the network structures.

**Figure 3:**
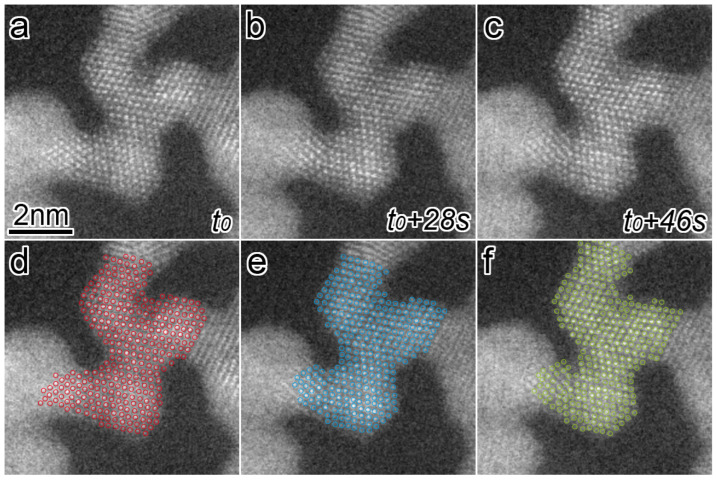
(**a**–**c**) Atomic-resolution HAADF-STEM images showing the structural change of Pt networks in air under the dose rate of 1.3 × 10^−1^ pA/nm^2^. The elapsed time is displayed at the right of each image. (**d**–**f**) Atomic columns in Pt networks in (**a**–**c**) are marked by red, blue, and green circles.

**Figure 4:**
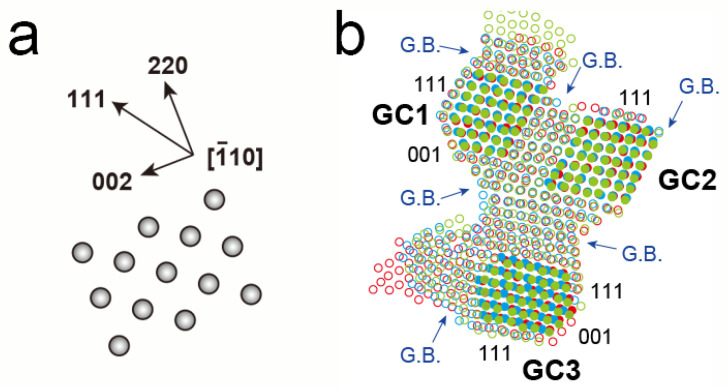
(**a**) Schematic drawing of an atomic model and the orientational relationship of the FCC structure viewed from $\left[\bar{1}10\right]$. (**b**) Overlayed image of only the colored circles from Figure 3d–f. Atomic columns with less movement in the grains are filled with the same colors as the circles. The group of color-filled circles can be regarded as stable grain cores, labeled as GC1, GC2, and GC3.

The morphology change became more remarkable as the magnification increased. Figure 3a–c shows a series of atomic-resolution HAADF-STEM images, revealing multiple connected grains, captured and cropped from a STEM movie (Video S2), of which the STEM magnification and dose rate were 10 million times and 1.3 × 10^−1^ pA/nm^2^, respectively. The elapsed time is displayed at the bottom right of each image. The Pt networks in this viewing area change slowly for 46 s. In Figure 3a–c, each bright dot corresponds to an atomic column in the grains and is marked by red, blue, and green circles in Figure 3d–f, respectively. Figure 4a is a schematic drawing of an atomic model showing the orientational relationship of the face-centered-cubic (FCC) structure viewed from $\left[\bar{1}10\right]$. In Figure 3a–c, the three grains show a clear atomic structure with <011> incidence, and they are connected with their {111} planes. Figure 4b shows an overlayed image of only the colored circles from Figure 3d–f. The change of atomic structure in the central part of each grain is smaller than that in other parts, such as the surfaces and GBs. In Figure 4b, atomic columns with less movement in the grains are filled with the same colors as the circles. The group of color-filled circles can be regarded as stable grain cores. In Figure 3d–f, the grain cores are labeled as GC1, GC2, and GC3. The crystallographic orientation relations between GC1 and GC2 are well-matched, whereas GC3 had a mirror image relation with them (i.e., a twin relation). GC1 and GC2 have three GBs, whereas GC3 has only two GBs. In Figure 3, all the images are displayed by aligning them so that the outlines of GC1 and GC2 match. The positions of not only the outlines but also internal atomic columns are consistent. Notably, they are stable, despite atoms in their GBs continuing to fluctuate. As for two GBs supporting GC3, the GB between GC2 and GC3 is a twin boundary, and another, the GB between GC3 and the grain on the left side of GC3, appears to contain a crystallographic mismatch and be strained because the grain is largely misaligned from the <011> incidence. Hence, GC3 was unstable, unlike GC1 and GC2, and its internal and surrounding atoms were more likely to fluctuate.

**Figure 5:**
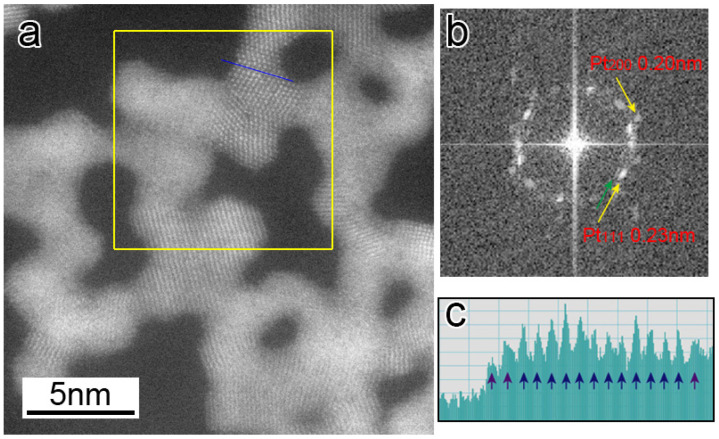
(**a**) HAADF-STEM image of the Pt networks captured from a STEM movie (Video S2). (**b**) FFT pattern calculated from a region inside a yellow rectangle in (**a**), in which diffraction spots corresponding to Pt {111} and Pt {200} planes are observed. (**c**) Intensity line profile along the blue line in (**a**).

In addition to the fluctuation of atoms at the GBs (i.e., unstable GBs) among GC1, GC2, and GC3, the atoms continuously rearranged on their surfaces. Figure 5a shows a HAADF-STEM image captured from a STEM movie (Video S2), and Figure 5b shows a fast Fourier transformation (FFT) pattern calculated from a region inside a yellow rectangle in 5a, in which diffraction spots corresponding to the Pt {111} and Pt {200} planes are observed. It should be noted that there are extra spots inside the Pt {111} spots. The radius of the extra spots from the center spot is approximately 10% smaller than that of the Pt {111} spots. Figure 5c shows an intensity line profile along a blue line in 5a, illustrating that the atomic spacing of surface layers is approximately 10% larger than that of the bulk ones. From Figure 5b,c, it is suggested that the lattice spacing of a few atomic layers of Pt {111} surfaces expanded by approximately 10% along the surface direction. Figure 4b (and Video S2) shows surface atoms repeatedly appearing and disappearing randomly, but no atomic step motion was recognized. This differs from the surface step growth reported in the case of catalytically reacting surfaces of NPG in a CO/air environment [17]. Yoshida et al. reported that the environmental TEM of Pt nanoparticles supported on cerium oxides under oxidation conditions showed the transformation of the nanoparticle surfaces to oxides layers, for which the lattice constant was larger than that of Pt, and the oxide layers grew further by increasing the oxygen partial pressure and electron beam intensity [35]. Since the formation of the surface layers with an expanded lattice spacing in our observation is consistent with their results, the surface layers covering the network structures may be an oxide phase.

The present observations showed that the morphology changes were mainly due to the movement of unstable grains, as shown in Video S1. Furthermore, no atomic step motion was noted, and neither surface growth nor etching was observed, although the atoms at the surfaces and GBs fluctuated, as shown in Video S2. Unstable grains and chained grains may deform or move to attach to neighboring parts in the network structures, and a GB-containing strain may attempt to relax its strain energy via atomic reconstruction or fracturing themselves. The connections of the grains through their {111} planes with small mismatches may be stable. Considering that the surface self-diffusion coefficients of a Pt atom on Pt {111} and an Au atom on Au {111} at 27 °C are calculated to be 5.3 × 10^−8^ and 8.3 × 10^−7^ cm^2^/s, respectively, using parameters in Ref. [36], diffusion-induced step motion of Pt atoms is intrinsically not likely to occur compared with that of Au atoms. In addition, oxygen molecules can be spontaneously dissociated and chemically adsorbed on Pt surfaces to be a surface oxide layer, while the chemisorption of oxygen on Au rarely occurs. Thus, differing from the case of NPG, the passive oxide surface on Pt in the present case hindered surface atomic diffusion, which explains why the coarsening and thinning of grains and GBs followed by the rupturing of connected chains barely occurred in the Pt networks.

## 4. Conclusions

Pt networks, which were composed of connected polycrystalline chains, were fabricated on the inner surface of a silicon nitride window in an EC, and their dynamic structural change was observed via atomic-resolution EC-STEM in air at 1 atm and room temperature. Owing to the shrinkage of the elongation along the beam direction of an aberration-corrected STEM probe with a wide convergence semi-angle, the SNR of the Pt network was enhanced, which is a dominant factor in improving the spatial lateral resolution, and atomic-resolution imaging was achieved. The exposure of Pt networks to the gas molecules stimulated by electron beams was considered to increase the collision probability between gas molecules and Pt networks, and the Pt networks are more intensively stressed from all directions than the situation without electron irradiation. The observations suggested that the morphology change of the Pt network in an oxidation environment was mainly attributed to the deformation and movement of chain-connected grains and GBs. Although atoms at the surface and GBs continued fluctuating, the surface atomic-diffusion-induced step motion was difficult to observe. Considering that our sample has two-dimensional network features, three-dimensional network catalysts that can be fabricated practically using a sophisticated design may show high stability because of the increased number of connection points among grains. By modifying our EC to enable the introduction of various gases, the technology can be expanded to more practical catalytic reaction experiments in the future. Aberration-corrected EC-STEM using a wide convergence angle is capable of improving the signal-to-background ratio of the samples in media sandwiched within the silicon nitride windows of an EC, opening the way to the in situ nano- and atomic-scale characterizations of catalysts in not only gases but also various kinds of liquids including electrolytes.

## Data Availability

Not applicable.

## Supplementary Materials

The following supporting information can be downloaded at: https://www.mdpi.com/article/10.3390/nano13152170/s1, Video S1: Takeguchi_S1.mp4; Video S2: Takeguchi_S2.mp4.
